# Anti-Candidal Activity of Reboxetine and Sertraline Antidepressants: Effects on Pre-Formed Biofilms

**DOI:** 10.3390/antibiotics12050881

**Published:** 2023-05-09

**Authors:** Eman Ibrahim Ahmed, Ahmed M. Alhuwaydi, Ahmed E. Taha, Mohamed Abouelkheir

**Affiliations:** 1 Department of Pharmacology and Therapeutics, College of Medicine, Jouf University, Sakaka 72388, Saudi Arabia; iimohamed@ju.edu.sa; 2 Department of Internal Medicine, College of Medicine, Jouf University, Sakaka 72388, Saudi Arabia; amalhuwaydi@ju.edu.sa; 3 Microbiology and Immunology Unit, Department of Pathology, College of Medicine, Jouf University, Sakaka 72388, Saudi Arabia; 4 Medical Microbiology and Immunology Department, Faculty of Medicine, Mansoura University, Mansoura 35516, Egypt; 5 Pharmacology Department, Faculty of Medicine, Mansoura University, Mansoura 35516, Egypt

**Keywords:** azoles, biomass, candidiasis, *C. albicans*, *C. glabrata*, *C. krusei*, fluconazole, itraconazole

## Abstract

Reboxetine (REB) and sertraline (SER) are antidepressants. The antifungal potential of these drugs against planktonic Candida has been recently reported with limited data about their effects on Candidal biofilms. Biofilms are self-derived extracellular matrixes produced by the microbial population that is attached to biotic surfaces, such as vaginal and oral mucosa, or abiotic surfaces, such as biomedical devices, resulting in persistent fungal infections. The commonly prescribed antifungals, azoles, are usually less effective when biofilms are formed, and most of the prescribed antifungals are only fungistatic. Therefore, the current study investigates the antifungal potentials of REB and SER, alone and in combination with fluconazole (FLC) and itraconazole (ITR) against Candidal biofilms. Using proper controls, Candida species (*Candida albicans*, *C. albicans*; *Candida krusei*, *C. krusei*; and *Candida glabrata*, *C. glabrata*) were used to form biofilms in 96-well microplates. Serial dilutions corresponding to concentrations ranging from 2 to 4096 µg/mL of the target drugs (REB, SER, FLC, ITR) were prepared and added to the plates. Impairment of the biofilm biomass and biofilm metabolic viability was detected using the crystal violet (CV) assay and 3-(4,5-dimethyl-thiazol-2-yl)-2,5-diphenyl tetrazolium bromide (MTT) assay, respectively. In the checkerboard assay, the sessile fractional inhibitory concentration index (SFICI) was calculated to evaluate the effects of drug combinations. SER was more effective in reducing the biomass than REB for *C. albicans* and *C. glabrata*, but both were equal for *C. krusei*. For the reduction in metabolic activity in *C. albicans* and *C. glabrata*, SER had a slight advantage over REB. In *C. krusei*, REB was slightly more potent. Overall, FLC and ITR were almost equal and produced more significant reductions in metabolic activity when compared to SER and REB, except for *C. glabrata*, where SER was almost equal to FLC. Synergism was detected between REB + FLC and REB + ITR against biofilm cells of *C. albicans*. Synergism was detected between REB + ITR against biofilm cells of *C. krusei*. Synergism was detected between REB + FLC and REB + ITR against biofilm cells of *C. albicans*, *C. krusei*, and *C. glabrata*. The results of the present study support the potential of SER and REB as anti-Candidal biofilm agents that are beneficial as a new antifungal to combat Candidal resistance.

## 1. Introduction

In the past few years, invasive fungal infection (IFI) incidence has shown a significant increase, with a mortality rate of around 90% [1,2]. Factors such as the use of immunosuppressant drugs, the uncontrolled use of broad-spectrum antibiotics, and the increased use of invasive procedures were incriminated [3]. Therapeutic options for fungal infections are limited. Azoles are the most used drugs for fungal infection [4]. While Candida is one of the most common pathogens in IFI [3], resistance of Candida to azoles has been increasingly reported [4]. Resistance to antifungals has become a major concern in clinical practice. Moreover, azoles are less effective against Candida when biofilms are formed [5].

Biofilms are self-derived extracellular matrixes produced by the microbial population that is attached to biotic surfaces, such as vaginal and oral mucosa, or abiotic surfaces, such as biomedical devices [6]. Biofilm formation is a well-known resistance mechanism formed by many bacteria [7,8] and fungi [6,9] in which they create more favorable conditions for their growth, and they become more difficult to eliminate than planktonic organisms. Biofilms result in the colonization of implanted devices that adversely affects their function, contribute to high antifungal resistance, and escape from host defenses, resulting in persistent fungal infections [9]. Within the biofilm, Candidal sessile cells have exhibited up to a 1000-fold reduced susceptibility to azoles [10].

Many antidepressants, mainly selective serotonin reuptake inhibitors (SSRIs), have shown antifungal activities. Over the past few years, SSRIs have been in focus as antifungal drugs [11,12,13,14]. The proposed mechanism for such antifungal activity is related to the modulation of the membrane and the vesicle-mediated transport or inhibition of fungal protein synthesis [15,16,17]. One SSRI, sertraline (SER), can reduce the cryptococcal burden in vitro and in the brain and kidneys [13,17]. However, SER is less effective against Candida species, and it was reported to antagonize the action of fluconazole (FLC) in vitro [17]. Many previous studies focused on the antifungal activities of SSRIs [11,12,13,14,17].

Reboxetine (REB) is a new antidepressant that works through a mechanism that differs from SSRIs. It acts by inhibiting norepinephrine reuptake [18]. Although the drug has secondary activities like SSRIs with the inhibition of P glycoprotein [19], only one simple in vitro screening study investigated its antibacterial and antifungal potential [16]. Kalayci and his colleagues [20] screened 16 different psychotropic drugs, including REB and SER, against a wide range of bacteria and fungi using a wide range of dilutions. They found that REB and SER could inhibit *Candida albicans* (*C. albicans*) growth in concentrations that could be achieved through dosage manipulation. Neither biofilm nor a combination with azoles was tested in this study. 

New drugs with antifungal effects should be investigated because of the toxicities, cost, and efficacy of the currently available antifungal drugs [2,21]. As antidepressants, REB and SER can reach the CNS and, if proven effective, can potentiate azole activities against Candida, affecting the CNS. The suspected benefits of the antifungal effects of REB and SER will be against not only Candida affecting the CNS but also cutaneous and mucocutaneous candidiasis, including vulvovaginal infections.

Limited data are available about the activity of REB and SER against different species of Candida and their drug interaction with different azoles, especially against Candidal biofilms. Therefore, the current in vitro study aims to investigate the antifungal potentials of REB and SER, alone and in combination with FLC and itraconazole (ITR), against Candidal biofilms.

## 2. Materials and Methods

### 2.1. Antifungal and Antidepressant Drugs

The antifungals (FLC and ITR) and the antidepressants (REB and SER) were obtained in powder form. Fresh solutions for the drugs were prepared before every experiment. According to the manufacturers’ instructions, dimethyl sulfoxide (DMSO) was used as a co-solvent to maximize powder solubility. Roswell Park Memorial Institute (RPMI) medium was used to prepare serial dilutions corresponding to concentrations ranging from 2 to 4096 µg/mL of the drugs. The reported REB planktonic MIC (128 µg/mL) and MFC (512 µg/mL) to *C. albicans* comprised the drug solution range to test its effect against Candidal biofilms [20]. Furthermore, the reported SER planktonic MIC (128 µg/mL) and MFC (128 µg/mL) to *C. albicans* comprised the drug solution range to test its effect against Candidal biofilms [20].

### 2.2. Candida Strains

*C. albicans* from American-Type Culture Collection (ATCC 10231), *C. krusei* (ATCC 6258), and *C. Glabrata* (ATCC 14053) were used. Stocks of each strain were kept frozen in brain heart infusion (BHI) broth with 5% glycerol at −80 °C until testing. In addition, one for each experiment, Yeast Peptone Dextrose (YPD) agar plates were used to subculture Candida isolates twice at 35 °C for 24 h in order to assess culture purity and viability [22].

### 2.3. Biofilm Formation

For biofilm growth, the method described by Ramage et al. [23] was followed. Briefly, Candida strains were allowed to grow overnight in YPD agar plates at 37 °C. The next step involved the inoculation of the products into YPD broth. The broth was then incubated overnight at 30 °C on a rotary shaker at 180 rpm. Centrifugation at 3000× *g* was used to harvest the Candida strains, which were then washed twice with 20 mL of sterile phosphate-buffered saline (PBS). A final concentration of 0.5 Macf (1 × 10^6^ cells/mL) was obtained by diluting the suspension in PPMI. The next step was transferring 100 μL of the diluted suspensions to 96-well microplates. Finally, biofilm growth was achieved by incubating the microplate at 37 °C for 48 h.

### 2.4. Biofilm Biomass Quantification

Measurements of the effects of REB and SER on the total biomass of pre-formed biofilms were conducted using the previously described crystal violet (CV) staining protocol [24]. The pre-formed biofilms were carefully washed twice with PBS. The next step was to add REB or SER in concentrations ranging from 2 to 4096 µg/mL to independent plates. These plates were then incubated for 24 h at 37 °C. In the negative control group, neither of the drugs was added. Additional controls in which DMSO was added were also included to exclude the possible effect of the organic solvent. After discharging the antidepressant solutions, the fixation of the biofilms was performed using 100 μL of 99% methanol, which was then allowed to dry in air. The following step was staining by the addition of 100 μL of a 0.02% CV solution. After 15 min, distilled water was used to remove the excess of CV. A volume of 150 μL of a 33% acetic acid solution was then added to release the bound CV. The absorbance was measured at 590 nm using a benchmark microplate reader. Experiments were performed in quadruplicates. The percent reduction in biofilm biomass for each drug-containing well was calculated in comparison with the biofilm biomass formed in the absence of any drug (growth control). The lowest concentration showing ≥50% reduction in biofilm biomass in comparison with the growth control was determined. 

### 2.5. Biofilm Metabolic Viability

Different concentrations of the antifungals (ranging from 2 to 4096 µg/mL) were prepared separately. A volume of 100 μL of each drug was transferred directly into the wells of independent plates to avoid disruption of the biofilms. Samples were then incubated at 37 °C for 24 h. Measurements of the effects of REB and SER on the metabolic activity of pre-formed biofilms were conducted using MTT as described by Pires et al. [25]. The pre-formed biofilms were carefully washed twice with PBS. The next step was to add REB or SER in concentrations ranging from 2 to 4096 µg/mL to independent plates. These plates were then incubated for 24 h at 37 °C. Controls were included in each plate with RPMI medium only (no added drug or film) and biofilm alone (no added drug; growth control). Additional controls in which DMSO was added were also included to exclude the possible effect of the organic solvent. After discharging the antidepressant solutions, we added a volume of 100 μL of the MTT solution (0.5 mg/mL) in PBS containing 0.1% glucose. The next step was the incubation of the plates in the dark at 37 °C for 6 h. After the removal of the MTT solution and washing biofilms once with PBS, the formazan product, re-suspended in acidic isopropanol, was added, and the absorbance was measured at 540 nm using a benchmark microplate reader. Experiments were conducted in quadruplicates. The minimal concentrations of REB or SER that were able to reduce the biofilm metabolic activity by 50% in comparison to the respective growth control (absence of drug) were defined as sessile cells minimal inhibitory concentration (SMIC_50_) [26]. 

### 2.6. Antifungal Activities of REB and SER in Combination with FLC and ITR on Candidal Biofilms

The initial fungal cells were set at 2 × 10^6^ CFU/mL. The tested concentrations ranged from 2 to 4096 µg/mL for REB, SER, FLC, and ITR. The minimal drug concentration that reduced the metabolic activity of the biofilm by 80% in comparison to the respective control (absence of drug) was defined as SMIC_80_ [27]. In the checkerboard assay, the sessile fractional inhibitory concentration index (SFICI) was calculated as [SMIC_80_-REB (or SER) in combination/SMIC_80_-REB (or SER) alone] plus [SMIC_80_-FLC (or ITR) in combination/SMIC_80_-FLC (or ITR) alone], in which synergism was interpreted as SFICI < 0.5, indifference was defined as 0.5 < SFICI < 4.0, and antagonism was SFICI > 4.0 [28].

### 2.7. Data Analysis

Data were analyzed using SPSS (ver. 22; SPSS Inc., Chicago, IL, USA). *t*-test (biomass) and ANOVA (metabolic activity) were followed by the Bonferroni post hoc test. Statistical significance was considered at *p* ≤ 0.05.

## 3. Results

### 3.1. Reduction in Biomass

**Figure 1:**
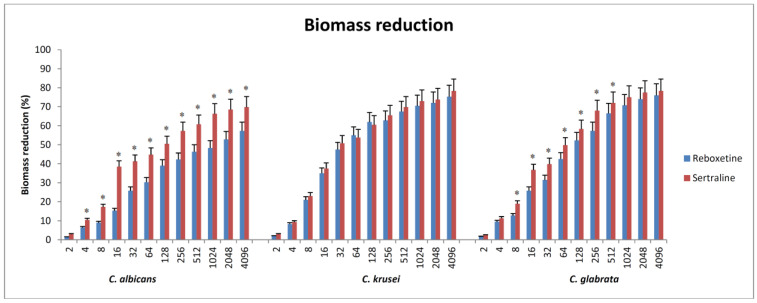
The relations between different concentrations of the tested drugs and the reduction in biomass. The percent reduction of biofilm biomass for each drug-containing well was calculated in comparison with the biofilm biomass formed in the absence of any drug (growth control). The lowest concentration showing a ≥50% reduction in biofilm biomass in comparison with the growth control was determined. For *C. albicans* and *C. glabrata*, sertraline showed significantly more reduction in biomass than reboxetine. For *C. krusei*, sertraline and reboxetine are equal. *C. albicans* appeared to be much more resistant to the effect of both drugs in comparison to the other species. * Significance versus reboxetine of the same concentration.

For *C. albicans* and *C. Glabrata*, SER resulted in significantly more reduction in biomass in comparison to REB. Both antidepressants were equal in the reduction in biomass of *C. krusei*. At 128 µg/mL, we noticed a reduction of at least 50% in the biofilm biomass of all species. One exception was REB, which was able to produce a 50% reduction in the biofilm biomass of *C. albicans* only at 2048 µg/mL. For the maximum tested concentration of SER (4096 µg/mL), all species showed significant reductions in biomass biofilm, which ranged from 69.8 % for *C. albicans* and 78.3% for *C. Glabrata* & *C. krusei*. Generally, it seems that the *C. albicans* strain was much more resistant to the effects of both drugs in comparison to the other species (Figure 1). 

### 3.2. Reduction in Metabolic Activity

**Figure 2:**
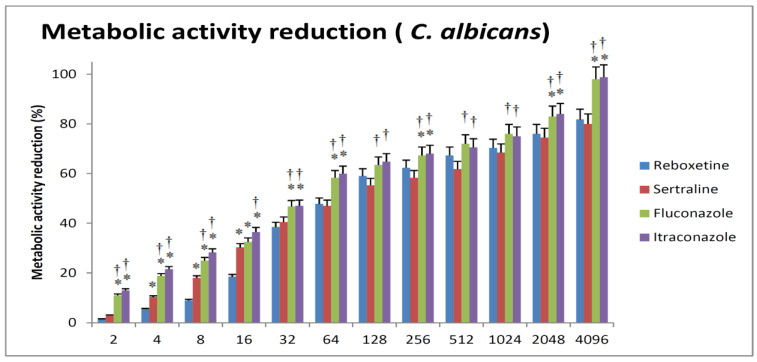
The relations between different concentrations of the tested drugs and the impairment of metabolic viability of *C. albicans*. The minimal REB or SER concentration that causes a 50% reduction in biofilm metabolic activity when compared with the respective growth control (absence of drug) is defined as sessile cell minimal inhibitory concentration (SMIC50). Sertraline has a slight advantage over reboxetine at lower concentrations. Fluconazole and itraconazole are almost equal and generally produce more significant reductions in metabolic activity when compared to sertraline and reboxetine. * Significance versus reboxetine of the same concentration. † Significance versus sertraline of the same concentration.

For *C. albicans*, SER resulted in more reduction in metabolic activity in comparison to REB at lower concentrations. Both drugs were able to reduce biofilm metabolic activity by 50% at about 128 µg/mL. When compared to FLC and ITR, both antifungals were equally effective and produced more significant reductions in metabolic activity when compared to SER and REB. FLC and ITR were able to achieve almost complete inhibition of metabolic activity at 4096 µg/mL, while SER and REB only achieved around 80% at the same concentration (Figure 2). 

**Figure 3:**
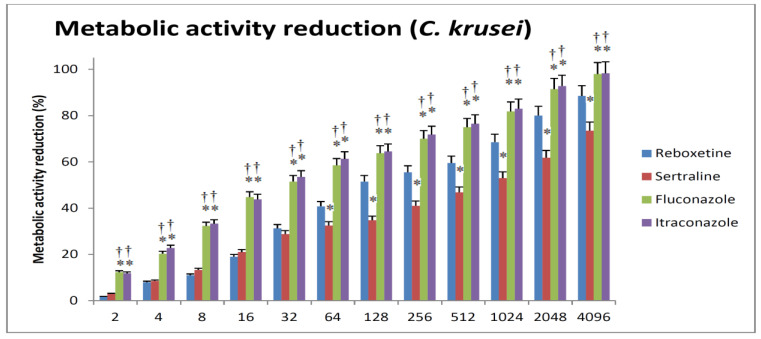
The relations between different concentrations of the tested drugs and the impairment of metabolic viability of *C. Krusei*. The minimal REB or SER concentration that causes a 50% reduction in biofilm metabolic activity when compared with the respective growth control (absence of drug) is defined as sessile cell minimal inhibitory concentration (SMIC50). Reboxetine produces more significant reduction of metabolic activity when compared to sertraline. Fluconazole and itraconazole are almost equal and generally produce more significant reduction of metabolic activity when compared to sertraline and reboxetine. * Significance versus reboxetine of the same concentration. † Significance versus sertraline of the same concentration.

For *C. krusei*, REB appeared to be more effective than SER in reducing metabolic activity at concentrations > 32 µg/mL. REB was able to reduce biofilm metabolic activity by 50% at about 128 µg/mL in comparison to 1024 µg/mL for SER. Again, both FLC and ITR were equally effective; Their potencies were higher than SER and REB. Both antifungals were able to achieve almost complete inhibition of metabolic activity at the highest concentration (Figure 3). 

**Figure 4:**
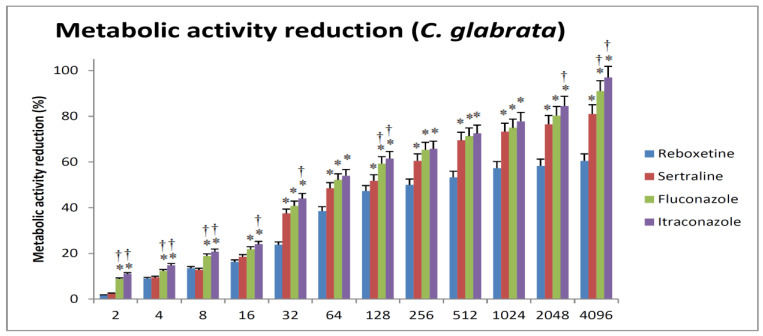
The relations between different concentrations of the tested drugs and the impairment of metabolic viability of *C. glabrata*. The minimal REB or SER concentration that causes a 50% reduction in biofilm metabolic activity when compared with the respective growth control (absence of drug) is defined as sessile cell minimal inhibitory concentration (SMIC50). Sertraline has a slight advantage over reboxetine at higher concentrations. Fluconazole and itraconazole are almost equal and generally produce more significant reduction in metabolic activity when compared to reboxetine. Sertraline generally produces reduction in metabolic activity that is more or less equivalent to that of fluconazole. * Significant versus reboxetine of the same concentration. † Significant versus sertraline of the same concentration.

Finally, SER resulted in significantly more reduction in metabolic activity in comparison to REB in *C. glabrata,* especially at higher concentrations. A reduction in biofilm metabolic activity by 50% was achieved at 128 and 256 µg/mL for SER and REB, respectively. SER was almost comparable to FLC in its ability to reduce metabolic activity (Figure 4).

Synergism (SFICI ≤ 0.5) was detected between REB + FLC and REB + ITR against biofilm cells of *C. albicans*. Synergism was detected between REB + ITR against biofilm cells of *C. krusei*. Synergism was detected between REB + FLC and REB + ITR against biofilm cells of *C. albicans*, *C. krusei*, and *C. glabrata* (Table 1 and Table 2) (Interpretation).

## 4. Discussion

Annually, there are over 1.5 million incidents of IFI with mortality rates that can reach as high as 90% [1]. The antifungal activities of new drugs should be investigated to avoid toxicities, as well as the cost versus efficacy of the currently available antifungal drugs [2,21]. In the present study, we were able to demonstrate that both SER and REB have some activity against three different strains of Candidal biofilms. Both drugs could reduce biofilm metabolic activity and enhance biofilm biomass disaggregation in a dose-dependent manner. Moreover, the ability of SER to inhibit the metabolic activity in *C. glabrata* was comparable to the classic antifungal, FLC. Furthermore, the present study tested the interactions between the SER and REB antidepressant agents on one side and FLC and ITR antifungals on the other side. No antagonism was detected between the tested agents. Synergism (SFICI ≤ 0.5) was detected between REB + FLC and REB + ITR against biofilm cells of *C. albicans*. Synergism was detected between REB + ITR against biofilm cells of *C. krusei*. Synergism was detected between REB + FLC and REB + ITR against biofilm cells of *C. albicans*, *C. krusei*, and *C. glabrata*.

The ability of certain antidepressants to inhibit fungal growth has been previously reported [11,12,13,14]. A recent study by Alkhalifa et al. (2022) reported that, among different SSRIs, SER outweighed fluoxetine, fluvoxamine, and paroxetine both as an antifungal and in enhancing the effect of FLC against *C. glabrata* strains. The FLC and SER combination was synergistic against even resistant strains known to express efflux pumps [29]. While almost all the studies tested the potential of SER, studies on REB were very limited in the literature. However, one study conducted by Kalayci and his colleagues [20] screened 16 different psychotropic drugs, including REB and SER, against a wide range of bacteria and fungi using a wide range of dilutions. They found that REB and SER could inhibit *C. albicans* growth in concentrations that could be achieved through dosage manipulation. 

Most of these studies did not test the antifungal activity of the antidepressant when the fungal biofilm is formed. Even the commonly prescribed antifungals, such as azoles, are usually less effective when biofilms are formed [5,10]. The re-evaluation of the antifungal-biofilm activity of antidepressants has drawn the attention of scientists over the last few years. In one study, it was reported that SER could reduce the biomass of *C. glabrata* by 88% and the biofilm metabolism of *C. parapsilosis* by about 90% [22]. Similarly, it was found that SER, alone or in combination with FLC, was superior to paroxetine and fluoxetine as an antifungal and antibiofilm against six isolates of four different Candida species [30]. 

To our knowledge, the present study was the first report on the antibiofilm activity of REB. Although SER was superior to REB in almost all the tests, we found that REB outweighed SER in reducing the metabolic activity in the *C. krusei* biofilm. Other than Candida, the efficacy of SER as an antifungal extends to different yeasts species, such as cryptococcus [17]. Attributing the variable effects of these antidepressants to the mechanism of action is somewhat difficult. Although we did not investigate the mechanism by which these two SSRIs inhibit Candidal biofilm, other studies suggested multiple targets in the fungal cell. In the earliest reports, it was suggested that SER use could reduce the production of phospholipase and aspartyl proteinase [31]. The modulation of the membrane and the vesicle-mediated transport, targeting phospholipids in the cytoplasmic surfaces, such as the Golgi apparatus or endosomes, or the inhibition of fungal protein synthesis have also been suggested [15,16,17]. The blocking effect of SER on the active efflux pumps in *C. glabrata* may also explain its synergistic effect with fluconazole [29]. Costa-Silva and his research team suggested that antidepressants can induce apoptosis in the fungal cells due to damage to the plasma and mitochondrial membranes [14].

Other than multiple targets of the tested antidepressants in the fungal cell, there is an additional factor that may also explain the superior antifungal effect of SER in comparison to REB. This additional factor is lipid solubility. The lipid solubility of SER is much higher than that of REB (log P = 5.15 vs. log P = 3.28, respectively) [32,33]. In one study, it was found that the parameter of lipophilicity, or lipid solubility, plays an important role in the antifungal activity of a class of experimental compounds [34]. The lipophilicity of the drug has a major role not only in determining the assimilation and distribution of that drug in living organisms but also in allowing the drug to penetrate the biofilm created by the fungal cell by destroying its matrix; it may also damage the plasmatic and mitochondrial membranes [22]. 

Clinically, antidepressant drugs are taken orally using a therapeutic dose for SER and REB of about 50–200 mg [35] and 4–12 mg [36] daily, respectively. However, knowing that the average plasma concentration for SER and REB is about 25 ng/mL and 169–663 ng/mL, respectively [37,38], it appears that these levels are significantly lower than the taken daily therapeutic dose. This can be explained by their extensive plasma protein binding (>97%) [22,36]. Considering several bioavailability and kinetics factors, we cannot use the results of our in vitro study to predict the in vivo performance of the selected antidepressants. Further in vivo studies are required to investigate the mechanism of the antifungal effects of SER and REB. Still, we can say that SER and REB are potential anti-Candidal drugs awaiting more characterization. 

## 5. Conclusions and Recommendations

The results of the present study support the potential of SER and REB as anti-Candidal biofilm agents that can be beneficial to counteract the antifungal resistance phenomena of Candida. Furthermore, while using SER and REB for the anti-Candidal purpose, their systemic effects against CNS, cutaneous, and mucocutaneous candidiasis must be determined, especially at high doses. Further studies are required in this regard and to determine whether their plasma-achieved concentration can be sufficient against mucocutaneous and cutaneous candidiasis and whether their plasma-achieved concentration can be beneficial against biofilms formed within medical devices such as intravenous catheters. We also need to compare the use of topical administration versus the oral intake of these drugs in the case of cutaneous or mucocutaneous candidiasis.

## Figures and Tables

**Table 1:** Interactions of reboxetine (REB) alone/in combination with fluconazole (FLC) and REB alone/in combination with itraconazole (ITR) against biofilm cells of Candida species.

Strains	SMIC_80_ Alone (µg/mL) ^1^			SMIC_80_ in Combination (µg/mL) ^1^		sFICI ^2^ of REB + FLC (Interpretation)	sFICI ^2^ of REB + ITR (Interpretation)
	REB	FLC	ITR	REB/FLC	REB/ITR		
*Candida albicans* (ATCC 10231)	>1024	>1024	>1024	64/256	64/256	0.3125 (synergism)	0.3125 (synergism)
*Candida krusei* (ATCC 6258)	>1024	1024	1024	512/512	64/128	1 (indifference)	0.1875 (synergism)
*Candida glabrata* (ATCC 14053)	>1024	>1024	>1024	512/512	512/512	1 (indifference)	1 (indifference)

^1^ The minimal drug concentration that caused an 80% reduction in biofilm metabolic activity when compared with the respective control (absence of drug) was defined as SMIC80. ^2^ The sessile fractional inhibitory concentration index (SFICI) was calculated as [SMIC80-REB in combination/SMIC80-REB alone] plus [SMIC80-FLC (or ITR) in combination/SMIC80-FLC (or ITR) alone], in which synergism was interpreted as SFICI ≤ 0.5, indifference was defined as 0.5 < SFICI < 4.0, and antagonism was SFICI ≥ 4.0.

**Table 2:** Interactions of sertraline (SER) alone/in combination with fluconazole (FLC) and SER alone/in combination with itraconazole (ITR) against biofilm cells of Candida species.

Strains	SMIC_80_ Alone (µg/mL) ^1^			SMIC_80_ in Combination (µg/mL) ^1^		sFICI ^2^ of SER + FLC (Interpretation)	sFICI ^2^ of SER + FLC (Interpretation)
	SER	FLC	ITR	SER/FLC	SER/ITR		
*Candida albicans* (ATCC 10231)	>1024	>1024	>1024	64/256	64/256	0.3125 (synergism)	0.3125 (synergism)
*Candida krusei* (ATCC 6258)	>1024	1024	1024	64/128	64/256	0.1875 (synergism)	0.3125 (synergism)
*Candida glabrata* (ATCC 14053)	>1024	>1024	>1024	256/128	256/128	0.375 (synergism)	0.375 (synergism)

^1^ The minimal drug concentration that caused an 80% reduction in biofilm metabolic activity when compared with the respective control (absence of drug) was defined as SMIC80. ^2^ The sessile fractional inhibitory concentration index (SFICI) was calculated as [SMIC80-SER in combination/SMIC80-SER alone] plus [SMIC80-FLC (or ITR) in combination/SMIC80-FLC (or ITR) alone], in which synergism was interpreted as SFICI ≤ 0.5, indifference was defined as 0.5 < SFICI < 4.0, and antagonism was SFICI ≥ 4.0.

## Data Availability

All datasets generated or analyzed during this study are included in the manuscript.
